# Decision making in vaccine hesitant parents and pregnant women – An integrative review

**DOI:** 10.1016/j.ijnsa.2022.100062

**Published:** 2022-01-15

**Authors:** Susan E. Smith, Nina Sivertsen, Lauren Lines, Anita De Bellis

**Affiliations:** a Flinders University College of nursing and health science, Australia; b Arctic University of Norway, Rural and Remote Arctic Health, Campus Hammersfest

**Keywords:** Vaccination, Vaccine refusal, Vaccine hesitancy, Antivaccination movement, Anti-vax, Pregnant women, Mother/father, Parent

## Abstract

**Objectives:**

**:** Vaccine refusal is increasing in Australia and is a major concern in high- and middle-income countries. There is evidence to suggest that some parents, even those who elect to immunise, may be vaccine hesitant with some manipulating the schedule by excluding or delaying some vaccines. The aim of this review was to gain an understanding of factors that influence vaccine decision-making in pregnant women and parents of children.

**Design:**

**:** An integrative review approach was used to produce an analysis of existing literature on vaccine decision-making in pregnancy and parents. As the broadest of review methods, an integrative review can include a range of experimental and non-experimental research, thereby ensuring the inclusion of data from multiple perspectives.

**Data Sources:**

**:** Online databases were searched for research related to vaccine decision-making in pregnant women and parents. Original and review articles were sought that were published in English between 2015 and 2021. Reviewed articles included qualitative and quantitative studies and systematic reviews. No mixed methods papers were located or excluded from this review.

**Review methods:**

**:** The review method was an integrative review informed by Coughlan.

**Results:**

**:** Papers from thirteen predominantly high- and middle-income countries were selected for this review. A total of 31 articles fit the inclusion/exclusion criteria, including qualitative, quantitative and review articles. Three main themes were identified including the role of healthcare professionals, vaccine safety concerns and alternative influences. Alternative influences included: social media, friends and family, religion, conspiracy theories and salutogenic parenting. Findings suggest that high levels of anxiety are involved in vaccine decision-making with parents seeking information from multiple sources including healthcare professionals, friends and family and social media.

**Conclusions:**

**:** Pregnancy is an ideal time to provide education on both pregnancy and childhood vaccinations. However, some parents reported dissatisfaction in their therapeutic relationships with healthcare professionals. As a result, parents can resort to their own information seeking, in the main via social media which has been linked to vaccine refusal. Additionally, some healthcare professionals report feeling inadequately prepared for the role of immunisation promotion and provision. Parental information seeking from non-traditional sources has been shown to result in the acquisition of misinformation, exposure to conspiracy theories, the inevitable loss of vaccine confidence and subsequent vaccine refusal.

What is already known?

• Vaccine hesitancy is increasing in middle- and high-income counties.
• Maintaining high levels of immunisation is vital for herd immunity.
• Nearly half of the Australian population experience vaccine hesitancy.

What this paper adds.

• Pregnant women and parents lack trust in healthcare professionals who report feeling inadequately prepared to promote and provide antenatal and childhood immunisation and further education is needed.
• Vaccine safety concerns are the major factor in vaccine refusal.
• Social media usage is linked to vaccine refusal.

## 1 Introduction

Immunisation is universally accepted as one of the most significant public health initiatives, with childhood immunisation alone responsible for saving 2–3 million lives each year. However, more deaths could be avoided with greater immunisation coverage (WHO, 2019a). Vaccine hesitancy or refusal to immunise children is a growing concern in middle to high income countries and has been defined as the reluctance or refusal to immunise, despite the availability of vaccines and an immunisation service (Larson, 2018; WHO, 2019). Vaccine hesitancy or refusal has recently overtaken vaccine access as the primary barrier to immunisation uptake (Larson, 2018). It is of such concern that vaccine hesitancy was included in the top ten threats to global health by WHO (World Health Organisation) in 2019 (WHO, 2019a). Maintaining high levels of immunisation is important to ensure herd immunity, which provides protection to people who are either too young or medically unable to be immunised (Logan et al., 2018). By achieving high levels of immunisation in a population, disease outbreaks are reduced, thereby offering protection to the entire community. Whilst childhood immunisation uptake is high in Australia, recently achieving 95.09% coverage for five-year-old children, there is significant uptake shortfall in some communities, as well as in the uptake of antenatal immunisations. This shortfall in immunisation uptake suggests varying degrees of vaccine hesitancy (Department of Health, 2021a).

Historically, the Australian government has strongly promoted childhood immunisation and currently provides funding for 17 diseases (AIHW, 2018). Since 1998 parents have been required to demonstrate that their child is fully immunised to access full family assistance payments (Berry et al., 2017). The rate of conscientious objectors (those who refuse vaccination on the grounds of freedom of thought, conscience, or religion) was recorded between 1999 and 2015 and this figure rose to 2% of the population in 2013 (Beard et al., 2016; Department of Health, 2021b). More recently, financial and social incentives have been introduced including ‘No Jab-No Pay’ and ‘No Jab-No Play’, the withholding of state payments and the exclusion of unimmunised children from kindergartens and child care centres, as well as the removal of the “conscientious objection” caveat in 2015 (SA Health, 2019). These changes have resulted in the increased uptake of immunisation to its current level (Berry et al., 2017). However, a small proportion of parents continue to refuse to immunise their children and these cases are often clustered in largely rural areas (Beard et al., 2016). Despite the government incentives, approximately 5% of five-year-old children remain inadequately vaccinated (Department of Health, 2019). This figure is higher for one-year old vaccinated children (94.85%) and two-year-old vaccinated children (92.55%) (Department of Health, 2021a). Additionally, uptake of antenatal immunisations remains suboptimal (Mohammed et al., 2018).

Internationally, health systems and vaccine confidence vary in developed countries with France and Portugal having low levels of vaccine confidence, whilst the UK, USA and Canada have high overall confidence (Kennedy, 2019, 2020). Uptake also varies in developing countries such as Malaysia and Indonesia (Syiroj et al., 2019; Rumetta et al., 2020). Vaccine hesitancy is not a new phenomenon, but a significant factor is thought to be the viral spread of misinformation by a small but active anti-vaccination movement which uses social media to influence vaccine decisions of parents (Larson, 2018; Rossen et al., 2019). Vaccine safety concerns have arisen from the spreading of misinformation (College of Physicians, 2020). This includes neurological damage caused by DTP (diphtheria, tetanus, and pertussis) vaccine in the mid 1970′s and allegations of autism resulting from MMR (measles, mumps, rubella) vaccine in the 1990′s. These studies were later discredited and found to be fraudulent (College of Physicians, 2020).

A recent Australian study suggested that nearly half of Australian parents had some concerns about childhood vaccinations. Additionally, parents who refuse or delay immunisations are more likely to have considered their options prenatally (Glanz et al., 2013; Danchin et al., 2018). Parents have expressed a desire for simple balanced information about all vaccines during pregnancy, including antenatal, post-natal and childhood immunisations (Glanz et al., 2013). However, some parents remain unconvinced of the safety of vaccines or the severity of vaccine preventable diseases and choose to delay or refuse routine immunisations. Additionally, healthcare professionals have reported feeling challenged by encounters with vaccine hesitant parents and many healthcare professionals believe they are inadequately prepared for these discussions (Berry et al., 2017; Smith et al., 2020).

The objective of this integrative review was to explore and analyse the literature describing the vaccine decision making of vaccine hesitant pregnant women and parents of pre-school children. The results of 31 independent studies were synthesized to gain a deeper understanding of the decision-making process, the influences at play, sources of information and why some parents are vaccine hesitant or refuse to immunise their children according to the recommended schedule.

## 2 Method

The framework developed by Coughlan (2017) was adopted to guide the integrative literature review. Additionally, the use of Critical Appraisal Skills Programme (CASP) tool has ensured the inclusion of quality articles (CASP, 2018; Whittemore and Knafl, 2005). The review has also included a variety of recent articles, using a combination of qualitative and quantitative approaches, chosen from thirteen countries, which have given the review the broadest possible focus. The purpose of an Integrative review is to provide clarity on health issues and to gain a new perspective of a topic (Coughlan, 2017). Integrative reviews have been described as the broadest type of review which provide a deeper understanding of the research problem (Whittemore and Knafl, 2005). This form of literature review supports evidence-based practice for nursing, midwifery, and healthcare professionals in general and has the capacity to re-frame thinking on a specific phenomenon of interest. By using explicit and systematic methods and incorporating multiple sources of evidence, rigour is enhanced, resulting in a greater understanding of the research problem with the potential to develop a knowledge base, identify a research gap and inform practice, policy, and future research (Whittemore and Knafl, 2005). The review question was: What are the reasons and sources of information for parents who hesitate or who refuse to be immunised antenatally or who refuse to immunise their children?

## 3 Results

**Fig. 1:**
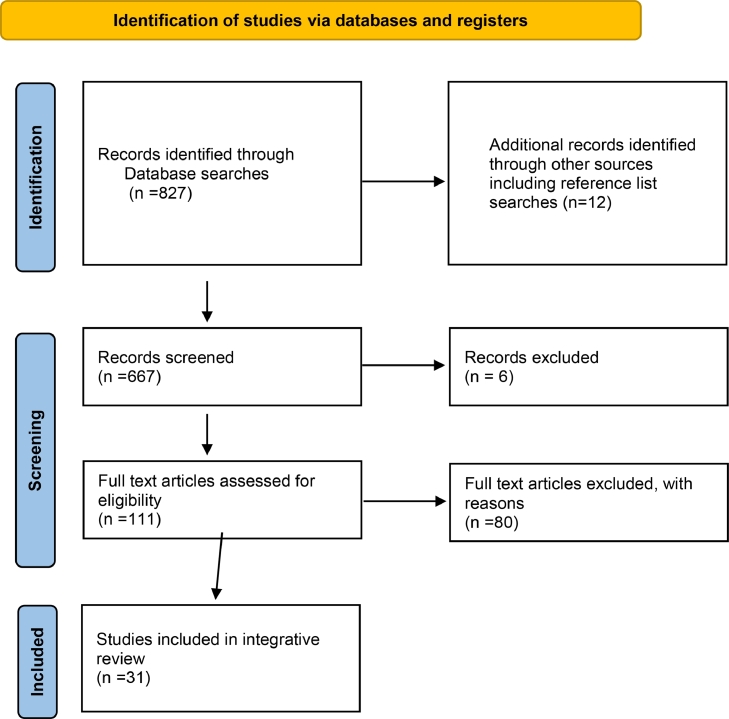
PRISMA flow diagram (Moher et al., 2015).

Upon definition of the research problem, a search strategy was designed in collaboration with a research librarian. The aim was to locate primary source articles with the inclusion/exclusion criteria identified (Fig. 1- PRISMA). To achieve an effective search strategy both a purposive approach and a comprehensive search of multiple databases were used. The following databases were identified as most appropriate as important sources of medical, nursing and immunisation literature: Medline; CINAHL; ProQuest; Scopus; and Web of Science. The inclusion criteria were decided upon, based on the most recent and appropriate articles available (Table 1 – Inclusion/Exclusion Criteria). Inclusion criteria were primary research studies, and systematic reviews including qualitative, quantitative and mixed methods studies which addressed the issue of vaccine decision making, vaccine hesitancy, vaccine refusal or schedule manipulation published between 2015 and 2020. English language was also an inclusion criterion. Reference lists were searched manually to ensure all pertinent papers were included. Studies with a focus on vaccines given in later childhood, such as Human Papilloma Virus (HPV) were excluded, as were papers focussing on vaccines such as Oral Polio Vaccine, Japanese encephalitis or other vaccines not included in the Australian schedule (Department of Health, 2020). This was done to ensure that the integrative review presented an Australian focus. The database searches were conducted electronically, and results were uploaded to Covidence for initial title and abstract screening by the principal author (Covidence Systematic Review Software, 2020). The following key words and phrases formed the basis of our literature search: # vaccination #vaccine # vaccine refusal # vaccine hesitancy # anti-vaccination movement # anti-vax # pregnant women # mother # father # parent.

**Table 1:** – Inclusion/exclusion criteria.

Inclusion Criteria	Exclusion Criteria
Primary research studies	Non-primary research papers
English language	Languages other than English
2015–2020	Studies conducted prior to 2015
Addressed the issue of decision making, vaccine hesitancy, schedule manipulation or refusal in pregnancy and parenting..	Studies with a focus outside early childhood immunisation, specifically Human papilloma Virus (HPV) or other vaccine provided to school aged children or adults.
	Papers with a focus on oral polio vaccine or other vaccine not included in the Australian schedule.

The search of the selected databases identified a total of 827 papers including 12 located in the manual search of reference lists. These papers were exported to Endnote X9 and subsequently, 161 duplicates were removed The EndNote Team 2013. A total of 666 potentially relevant papers were accepted for title and abstract screening and these were uploaded to Covidence in June 2020. After initial title and abstract screening by two reviewers (SS and NS), 111 papers were accepted for full screening and 80 were excluded at this point, subject to the search criteria. Finally, 31 papers were selected for inclusion in the review. These papers were checked for relevance, and all had ethics approval where appropriate (Fig. 1-PRISMA). The decision to include or exclude data was informed by both the research question and the selection criteria and maintained a strong focus on the decision-making processes of pregnant women and parents regarding their decision to not vaccinate their children (Table 2 -Review Table of Articles).

**Table 2:** – Review Table of Articles.

Authors	Study Aims	Study design and methods	Sample and setting	Findings	Limitations
(Attwell et al., 2018)	To explore how complementary and alternative medical practitioners effects vaccine decision making in *n* = 29 parents from South Australia and Western Australia.	Qualitative study.	Parents of children (*n* = 29) under five years.	This study reported that the use of complimentary and allied medical practitioners were not a preplacement for immunisation but a buttress for child health. Complimentary and allied medicine use did not cause vaccine hesitancy or rejection.	Findings cannot be generalised to the entire vaccine hesitant and rejecting population in Australia. Other limitations relate to the design and conduct of the study which were not conducted with complimentary and allied medicine focus.
(Ben Natan et al., 2017)	To identify factors associated with the intention to receive Pertussis vaccine in pregnancy in Israel (*n* = 220).	Quantitative survey.	Pregnant women native born in the former Soviet Union resident in Israel (*n* = 220).	Healthcare professionals (HCP) need to take a more active role in educating pregnant women on the risks of pertussis. Perceived risks and benefits of vaccines predicted intention to immunise.	Snowball sampling method can limit generalizability of study. Most participants were primigravida with academic education.
(Betsch et al., 2018)	To assess how and why vaccine attitudes change over time and what influences this change. Participants from Germany (*n* = 351, *n* = 204, *n* = 215) and (*n* = 173).	A prospective cohort study conducted during pregnancy, +3/12, +6/12 and + 14/12 postpartum.	Randomly assigned web-based study (*n* = 351, *n* = 204, *n* = 215) and (*n* = 173).	Maternal attitudes to immunisation are formed in their child's first year. Strategies should aim to improve experience during first vaccinations	Selection bias is a potential limitation as only women with an interest in health were recruited.
(Bradshaw et al., 2020)	To analyse social media content to determine how first-time pregnant and new mothers were treated and influenced by anti-vaccine advocates in a closed face-book group.	Qualitative discourse analysis.	A Facebook group with more than 100,000 members was the focus of this study.	Anti-vaccination advocates impacted first-time mothers’ intentions through influential and normative processes.	Paraphrasing of posts may be limitation. Also, study cannot be generalised as any qualitative study.
(Clarke et al., 2019)	To investigate the vaccine decision making process throughout pregnancy in the UK (*n* = 182).	Quantitative online study.	Pregnant women. during early and late pregnancy (*n* = 182).	88% reported seeking additional information about pertussis during pregnancy. Risk associated with pertussis vaccine decreased as pregnancy progressed.	Self-selection bias relating to participation in this study with higher than national average immunisation rate amongst participants.
(Costa-Pinto et al., 2018)	To determine the prevalence of vaccine concerns, socio-economic status, and vaccine uptake in Australian parents (*n* = 311) (*n* = 391).	A quantitative survey.	Parents of children under five years. attending general paediatric out-patients department and maternal child health centres in two Australian states (*n* = 311) (*n* = 391).	Nearly half of Australian parents have some concerns about childhood vaccines. HCP are best placed to provide education and address concerns.	Exclusion of non-English speaking parents may limit generalisability. Also limited access to vaccine hesitant parents is a limitation.
(Danchin et al., 2018)	To ascertain vaccine information received, maternal immunisation uptake and attitudes and concerns regarding childhood immunisation in Australia (*n* = 490, *n* = 295, *n* = 399 and *n* = 231).	Quantitative survey.	Pregnant women attending antenatal appointments at four public hospitals across four Australian states (*n* = 490, *n* = 295, *n* = 399 and *n* = 231).	First time mothers are more vaccine hesitant and only 2/3 of mothers believed they received enough information during pregnancy.	Only 43% of mother agreed to follow up. Only English-speaking mothers and low levels of indigenous mothers were included in the study.
(Diaz Crescitelli et al., 2020)	To conduct a systematic review and meta-synthesis of qualitative studies to identify key elements involved in childhood vaccine hesitancy.	Meta-synthesis.	A meta-synthesis was conducted on *n* = 27 qualitative papers from eight countries.	HCP need to address vaccine hesitancy. HCP need a better understanding of vaccine hesitancy to achieve effective communication.	A large number of studies from Western settings were included and none from Asian settings making the results more relevant to a Western setting.
(Dube et al., 2016)	To better understand why Canadian mothers (Quebec), choose to vaccinate – or not – their newborns (*n* = 56)	Qualitative interview.	Pre- and post-natal, of women (*n* = 56) were purposively recruited to achieve a balance of vaccine hesitant, vaccine refusers and vaccine accepters.	Many factors influence vaccine decision-making with many parents ambivalent about immunisation and continuing to question their decision.	Selection bias may exist as participants were voluntary. Generalizability is limited due to the nature of the study and the settings.
(Dube et al., 2018)	To explore vaccine hesitancy amongst Canadian parents and to examine factors associated with parents’ intention to vaccinate (*n* = 2013).	Quantitative survey.	Canadian parents of children under five years (*n* = 2013).	Parents information needs and searches as well as trust in institutions were associated with decision to vaccinate.	Selection bias due to participants having certain characteristics. Some study design issues including timing of participation.
(Duchsherer et al., 2020)	The aim of this study was to evaluate testimonials on a film entitled VAXXED, an anti-vaccination film.	Qualitative study	Testimonials on a film entitled VAXXED, an anti-vaccination film.	Distrust of doctors, self-diagnosis, building credibility evident in the content analysis.	Analysis limited to spoken word videos and testimonials. Findings cannot be generalized due to sampling and design of the study.
()	To conduct a survey of Italian parents to estimate vaccine hesitancy and its determinants (*n* = 3130.	Quantitative survey.	Parents (*n* = 3130) were surveyed to evaluate attitudes and beliefs to tetanus and measles vaccines	Vaccine safety is a concern by all parents but more so by vaccine hesitant parents. HCP need further training to respond to concerns.	Families residing in the north of Italy were over-sampled. Hesitancy rate could be over or under-estimated.
(Gidengil et al., 2019)	To conduct a systematic review of the literature to identify the range of beliefs around childhood vaccines.	Systematic Review.	A systematic review of 71 articles.	Concerns about vaccine safety were the most commonly stated beliefs about childhood vaccines.	The studies included in this review focussed on identifying barriers to immunisation. Findings are likely to be biased to negative findings about immunisation.
(Helps et al., 2019)	To explain vaccine refusal in a sample of Australian parents (*n* = 32).	Qualitative study.	Semi-structured interviews were conducted on parents (*n* = 32) and one pregnant woman.	Common patterns included perceived deteriorating health in Western society. Dismissive relationships with HCP. A quest for truth in ongoing risk assessment.	Interviews occurred in geographical cluster, Byron Shire. Study not generalizable.
(Helps et al., 2018)	To interview vaccine hesitant parents in the Byron Shire, Australia to ascertain the impact of legislation aimed to enhance vaccine compliance (*n* = 31).	Qualitative study.	Vaccine hesitant parents’ (*n* = 31) resident in the Byron Shire, a northern NSW coastal area.	No Jab No Pay legislation has resulted in greater commitment to not vaccinate, financial hardship and increased desire for control over health choices.	Unique geographical and social location of target group. Study not generalizable.
(Jenkins and Moreno, 2020)	To analyse how parent vaccination opinions are expressed online.	Qualitative study	Comments on parenting blogs related to vaccination. Nine blogs were included from 244 comments.	A high percentage of “attack” comments and inaccurate information evident in blog comments.	Comments on blog favoured pro-vaccination stance. Sample size small and aimed at most popular blogs, not anti-vaccination blogs.
(Koski and Holst, 2017)	To explore vaccine hesitancy in the Netherlands and Finland through an artist-scientist collaboration (*n* = 6).	Qualitative study.	Vaccine hesitant parents’ resident in either Finland or Netherlands (*n* = 6).	Diagrams and narratives merged to reveal health beliefs behind vaccine hesitancy.	Recruitment of vaccine hesitant parents through authors own social network was a limitation to the study.
(Lama et al., 2020)	To explore the predictors of childhood influenza status based on adult status in the United States of America (*n* = 328).	Quantitative study.	Survey of non-Hispanic black and white parents (*n* = 328).	Different approaches should be taken when educating vaccine hesitant and non-hesitant parents about childhood influenza vaccination.	Limitations exist in the sampling and the nature of the cross- sectional survey which provides only a snapshot in time of vaccine decision-making.
(Peretti-Watel et al., 2019)	To compare two populations with contrasting socio-economic profiles to evaluate vaccine hesitancy in France (*n* = 25).	Qualitative study.	Interviews (*n* = 25) of French parents of young children.	Despite the rise in use of the internet, participants also relied on face-to-face interactions with peers. Most trusted their own HCP.	Recall bias and social desirability bias are limitations in this study.
(Romijnders et al., 2019)	To investigate factors at play in informed vaccine decision making of childhood immunisation in the Netherlands (*n* = 12).	Qualitative study.	Three focus groups (*n* = 12) conducted in the Netherlands with vaccine acceptors, refusers, and partial acceptors.	Vaccine refusers and partial acceptors actively weighed pros and cons of vaccine, but knowledge not always evidence based.	Selection bias with high proportion (96%) highly educated.
(Rossen et al., 2019)	To examine the structure and roots of anti-vaccination attitudes, intentions, and moral preferences in Australia (*n* = 296).	Quantitative survey.	Parents or caregivers (*n* = 296) who were visitors to parenting websites and Facebook pages.	Compared to accepters, fence sitters and rejecters exhibited a moral preference for liberty and purity.	Australian parenting website data may not generalise to a broader population. Sample is self-selected and subject to bias.
(Rosso et al., 2020)	To conduct a systematic review of studies that assessed the knowledge and attitudes of pregnant women to paediatric vaccinations (*n* = 16).	Systematic review	Sixteen primary source articles were reviewed.	Pregnant women overall believe vaccines are important to protect their children. Vaccine safety concerns persist which reduce trust in vaccines.	Limitation are the nature of vaccine hesitancy itself which has been described as a context specific phenomenon. Survey may not be generalizable.
(Rozbroj et al., 2020)	To gain a deeper insight into the way having children influences vaccine beliefs of Australian parents (*n* = 904).	Qualitative study.	Australian parents (*n* = 904) who had indicated that they had changed their attitude to vaccination after having children.	Having children prompted parents to learn about vaccines. Hesitant parent distrustful of pharmaceutical companies fuelling fears of vaccine safety.	Paper focussed mainly on parents whose vaccine attitude remained unchanged by having children.
(Rumetta et al., 2020)	To explore Malaysian parents’ reasons for vaccine refusal and to report their views on recommendations on discussing vaccine-related concerns (*n* = 14).	Qualitative study.	Face to face and online in-depth interviews of (*n* = 14) parents who had refused any childhood vaccine.	Parents wanted more empathy from HCP and evidence of vaccine safety and purity.	Participants had a background of tertiary education and lacked representation of lower educated parents. These findings are not generalisable as sample was small.
(Saada et al., 2015)	The aim of this study was to gain a better understanding of parents’ rationales for their vaccine choices in USA (*n* = 24).	Qualitative study.	Semi-structured interviews of (*n* = 24) parents attending a health centre.	Parents who adopted alternative schedules wanted more control over exposure to vaccine ingredients, vaccine safety and held concerns over vaccine safety and necessity.	Sample was small and select and included only insured members of a health organization. Results cannot be generalised.
(Swaney and Burns, 2019)	The aim of this study was to explore reasons for vaccine hesitancy amongst higher socioeconomic parents in Perth WA (*n* = 18).	Qualitative study.	High income parents (*n* = 18) in WA who had concerns about vaccinating their children.	Parents believed they could make good vaccination decisions based on higher education and self-sourced information. Parents expressed concern over vaccine contents and expressed a low value of the benefits of herd immunity.	No limitations to this study were reported however, this research is not generalizable due to the nature of qualitative research.
(Syiroj et al., 2019)	The aim of this study was to explore parents underlying reasons for their child's incomplete immunisation in Indonesia (*n* = 16).	Qualitative study.	Semi structured interviews were conducted with parents (*n* = 16) of under immunised children in Banten Province.	Islamic beliefs, belief in natural immunity and the use of alternative medicine strongly influenced vaccine choices. Safety concerns and lack of trust in government as well as trust in information found on the internet also influenced vaccine choices.	Limitations include generalisability. The views of vaccine hesitant parents who subsequently vaccinate their children are not represented.
(Tomljenovic et al., 2020)	The aim of this study was to explore factors that contribute to parental vaccine conspiracy theories in Croatia (*n* = 823).	Quantitative study.	Explore Croatian parents (*n* = 823) reasons for incomplete immunization of their child.	Greater vaccine conspiracy beliefs were associated with unpleasant emotions towards immunisation. Intuitive thinking was also linked to vaccine refusal.	The data obtained were correlational and cannot be linked to any causal effect. A biased sample of mostly female participants from similar background also infer bias.
(Tustin et al., 2018)	The aim of this study was to investigate the link between parental perceptions of vaccine risk with seeking information from the internet in Canada (*n* = 966) (*n* = 951).	Quantitative study.	Facebook survey compared with data obtained from random digit dialling of Canadian parents by telephone survey. (*n* = 966) (*n* = 951).	The use of internet for vaccine information resulted in parents finding vaccines less safe than parents who did not use the internet.	The method of Randomised digit dialling is a limitation in this study as fewer people retain a landline thus compromising the samples representativeness.
(Wang et al., 2015)	The aim of this study was to examine how attitudes and beliefs are developed and contribute to immunisation decisions in USA (*n* = 23).	Qualitative study.	Interviews conducted on parents from the USA (*n* = 23) claiming to be pro-vaccine.	Parents were often overwhelmed with the quantity and ambiguity of information and perceived minimal consequences with deviating from the recommended schedule.	Sample populations were already interested in vaccination issues. The sample were also predominantly pro-vaccine and results cannot be generalised.
()	This paper aimed to explore the ways parents talked about perceived risks and benefits of vaccination in Australian parents (*n* = 29).	Qualitative study.	Interviews of non- vaccinating or vaccine hesitant Australian parents (*n* = 29).	Parents engaged in ongoing information seeking whilst using reason, rejection of western medicine, and salutogenic parenting to reduce exposure to toxins.	Data was analysed from two separate studies undertaken by two different researchers in two cities. Results are not generalisable due to nature of qualitative research.

This review evaluated 31 predominantly primary source articles, three systematic reviews and three content analyses of Facebook pages, parenting blogs and testimonials of the movie Vaxxed, published between 2015 and 2020. Data synthesis was achieved using descriptive coding to further organise data and to systematically compare and synthesise the findings of all studies. These codes were discussed amongst all authors and the final themes were agreed upon. Papers from thirteen predominantly high-income countries, were included in the review. Articles were also included from medium and low-income countries such as Malaysia and Indonesia, to provide a thorough understanding of global vaccine hesitancy. Nine articles were included that had an Australian focus, as well as seven articles from the United States of America, three papers from Canada and three papers from Italy. Papers were also included from Israel, United Kingdom, Germany, France, the Netherlands, Croatia and Finland(Table 3).

**Table 3:** – Studies contributing to the findings.

**Finding**	**Sources**
Healthcare professionals – Role and information provision	(Saada et al., 2015; Wang et al., 2015; Ben Natan et al., 2017; Costa-Pinto et al., 2018; Danchin et al., 2018; Betsch et al., 2018; Clarke et al., 2019; Helps et al., 2019; Peretti-Watel et al., 2019; Romijnders et al., 2019; Gidengil et al., 2019; Diaz Crescitelli et al., 2020; Duchsherer et al., 2020; Lama et al., 2020; Rosso et al., 2020; Rozbroj et al., 2020; Rumetta et al., 2020.
Vaccine safety concerns	(Wang et al., 2015; Ben Natan et al., 2015; Dube et al., 2016; Betsch et al., 2018; Cost Pinto et al., 2018; Danchin et al., 2018; Tustin et al., 2018; Ward et al., 2018; Clarke et al., 2019; Gidengil et al., 2019; Helps et al., 2019; Romijnders et al., 2019; Swaney and Burns, 2019; Diaz Crescitelli et al., 2020; Jenkins et al., 2020.
Alternative influences	(Dube et al., 2016; Koski and Holst, 2017; Attwell et al., 2018; Costa-Pinto et al., Danchin et al., 2018; Dube et al., 2018; ; Helps et al., 2018; Tustin et al., 2018; Clarke et al., 2019; Gidengil et al., 2019; Helps et al., 2019; Peretti-Watel et al., al.,2019; Rossen et al., 2019; Swaney and Burns, 2019; Syiroj et al., 2019; Bradshaw et al., 2020; Diaz Crescitelli et al., 2020; Duchsherer et al., 2020; Jenkins and Merino, 2020; Rozbroj et al., 2020; Rumetta et al., 2020; Tomljenovic et al., 2020.

A decision was made to undertake an inductive analysis as opposed to a theoretical thematic analysis. Inductive analyses code data without trying to fit it into a pre-existing pattern. Whereas, theoretical coding is driven by a desire to make the data fit a theoretical or analytical interest (Braun and Clarke, 2006). This decision was based on a strong desire to accurately reflect the content of the data. Therefore, the thematic analysis was driven by the data in line with the analytical framework of Braun and Clarke (2006). Three main themes were identified from the analysis including: healthcare professionals- role and information provision, vaccine safety concerns, and alternative influences on vaccine decision-making including: complementary and allied medical practitioners (CAM), conspiracy theories, the influences of friends and family, religion, social media and salutogenic parenting (Merriam-Webster.com, S. "Salutogenesis 2010) (Table 3 Studies contributing to the findings). Manual coding was used to identify the themes. The three major themes were: (i) vaccine safety concerns;  (ii) healthcare professionals’ role and information provision; and (iii) alternative influences on vaccine decision-making (see Table 4).

**Table 4:** – Themes and sub-themes.

**Major themes**	**Vaccine safety concerns**	**Healthcare professional's role and information provision**	**Alternative influences on decision-making**
Sub-themes	Long-term side-effects	Support and trusting relationships	Complimentary therapies and allied health professionals
	Conspiracy theories	Concerns and external influences	Internet and social media
	Risk versus benefits debate	Alternative schedules	Religion
		Inadequate preparation	Friends and family
		Information and education provision	Salutogenic parenting
		Poor communication	

### 3.1 Vaccine safety concerns

This theme highlighted the influence of safety concerns on vaccine decision making in the target population. Some of the specific concerns discussed in the literature included adverse reactions, vaccine contents and lack of purity (Rumetta et al., 2020; Swaney and Burns, 2019; Syiroj et al., 2019). Concerns also extended to fear of long-term side-effects including autism as well as auto-immune diseases (Gidengil et al., 2019). Evidence exists that high immunisation levels did not always imply high levels of vaccine confidence (Mendel-Van Alstyne, Nowak, and Aikin, 2018; Wang et al., 2015). Many factors influenced the decision to accept or reject vaccines and concerns about vaccine safety, which were present in both high- and middle-income countries, was one of the most cited reasons for vaccine refusal (Kumar et al., 2016; Kennedy, 2020). These included the perceived safety of the vaccine and the perceived low risks associated with diseases (Ben Natan et al., 2017; Clarke et al., 2019; Gidengil et al., 2019; Diaz Crescitelli et al., 2020). A meta-analysis conducted in Italy reported that parents often focussed more substantially on the risks associated with vaccines (Rosso et al., 2020). This was supported by studies in many other countries including Germany, Canada and the Netherlands, amongst others (Dube et al., 2016; Betsch et al., 2018; Costa-Pinto et al., 2018; Danchin et al., 2018; Romijnders et al., 2019; Jenkins and Moreno, 2020). Vaccine concerns were also cited in an Australian study of high-income parents, which reported low risk perception of vaccine preventable diseases and a disproportionately high belief of risks associated with vaccines (Swaney and Burns, 2019). Additionally, there was a higher level of vaccine concern associated with new vaccines such as pneumococcal and rota-virus vaccines reported (Gidengil et al., 2019). Safety concerns associated with vaccines have been shown to adversely affect vaccine choices. This, associated with the perception of low risk associated with vaccine preventable diseases, makes this a major theme in the review.

Vaccine safety concerns were one of the main reasons that parents become hesitant about immunisation (Gidengil et al., 2019; Saada et al., 2015). It is an area of global concern in both high, medium, and low-income countries (Syiroj et al., 2019; Rumetta et al., 2020). Additionally, high immunisation levels do not always imply high levels of vaccine confidence (). There is evidence that nearly half of Australian parents have some concerns about vaccines yet still ultimately immunise their children (Danchin et al., 2018). A recent Australian study has argued that vaccine hesitant parents consider conducting risk assessments their personal responsibility and questioning vaccines a part of that ().

The vaccine decision-making process is complex and often takes place over time and for some parents continues throughout their child's early years. Educating parents about the risks associated with vaccine preventable diseases can also present a challenge to healthcare professionals with both minimal immunisation education and limited experience of the diseases themselves. In comparison, the evidence presented by anti-vaccination groups are both emotive, and convincing.

### 3.2 Healthcare professionals – role and information provision

This theme showed the important role of healthcare professionals as the primary immunisation information source to pregnant women and parents. The role of the healthcare professional in the promotion and provision of immunisation was well established (Facciola et al., 2019; Kennedy, 2020). The recommendation of a healthcare professional was a predictor for immunisation uptake (Van Buynder, Van Buynder, Menton, Thompson, and Sun, 2019). This concept was supported by many studies in this review (Ben Natan, El Kravchenko, Sakashidlo, and Mor, 2017; Costa-Pinto et al., 2018; Danchin et al., 2018; ; Lama et al., 2020). Reports of between 62% and 67% of parents in the UK and USA respectively, chose to accept vaccines because of the information they received from a healthcare professional (Clarke et al., 2019; Lama et al., 2020). A recent Italian study, a country with high levels of vaccine safety scepticism, reported that healthcare professionals played a key role in informing parents about vaccines (). In terms of timing for this information, it was reported that during pregnancy was an optimal time to provide childhood vaccination education (Betsch et al., 2018; Danchin et al., 2018; Clarke et al., 2019;). Evidence suggested that parents and pregnant women placed trust in healthcare professionals to provide timely accurate and in-depth immunisation information (Krishnaswamy et al., 2018). However, this information was not always provided, and concerns were often overlooked or ignored (Costa-Pinto et al., 2018; Peretti-Watel et al., 2019; Romijnders et al., 2019; Rozbroj et al., 2020; Saada et al., 2015). Parents reported having their information needs ignored by healthcare providers and their concerns dismissed (Ben Natan et al., 2017; Helps et al., 2019). The importance of healthcare professionals in information provision and education of pregnant women and parents was well supported in the literature.

Similarly, a lack of trust in healthcare professionals was also identified in several studies. (Gidengil et al., 2019; Rumetta et al., 2020; Diaz Crescitelli et al., 2020; Duchsherer et al., 2020). A common criticism of healthcare professionals was their perceived dismissal of concerns, thereby, undermining trust in both the healthcare professional and the healthcare system in general (Helps et al., 2019; Rumetta et al., 2020). Parents also felt that healthcare professionals were influenced by vaccine manufacturers and therefore could not be trusted (Rumetta et al., 2020). Studies conducted in the USA reported the use of many alternative vaccine schedules, some with manipulated timing and others with vaccines excluded completely (Saada et al., 2015). One study identified five alternative schedules in use across one health maintenance organisation in Northern California. This accepted use of alternative schedules, whilst possibly designed to meet the needs of vaccine hesitant parents, may also be interpreted as justification of vaccine hesitancy, and ultimately lead to loss of confidence in immunisation (Wang et al., 2015). The lack of trust in healthcare professionals and their perceived dismissal of vaccine concerns demonstrated a need for focussed education in communicating appropriately with vaccine hesitant parents.

Healthcare professionals reported finding conversations with vaccine hesitant parents challenging and have also reported feeling inadequately prepared to promote and provide antenatal immunisation (Leask et al., 2012; Glanz et al., 2013; Berry et al., 2017; Facciola et al., 2019; Smith et al., 2020). There was evidence to suggest a need for additional education of healthcare professionals to promote effective communication with vaccine hesitant parents. Several studies in this review reported that healthcare professionals may need additional training to assist parents with vaccine decision making (; Helps et al., 2019; Rosso et al., 2020; Rumetta et al., 2020). This review demonstrated a knowledge gap in the ability of healthcare professionals to effectively communicate with vaccine hesitant parents.

Vaccine decision making has been reported to begin in pregnancy. Several studies reported that parents want more information on the risks and benefits of both pregnancy and childhood vaccines during pregnancy (Ben Natan et al., 2017; Betsch et al., 2018; Danchin et al., 2018). Additionally, one large German study (*n* = 1299) reported that the vaccination experiences and the information received in the first year of life, are critical to vaccine decision making (Betsch et al., 2018). Several studies described the important role of midwives in antenatal immunisation education and provision (Danchin et al., 2018; Rosso et al., 2020). Pregnancy is a time when effective communication of the risks and benefits of immunisation are vital. This is also an opportunity to demonstrate understanding of the concerns of vaccine hesitant parents whilst addressing each concern calmly and respectfully thereby building the therapeutic relationship.

Parental lack of awareness about vaccination was cited as a reason for vaccine hesitancy (Dube et al., 2018). This was supported by an Italian review of the literature, which claimed it was the main reason for vaccine hesitancy (Rosso et al., 2020). Additionally, a UK study reported that perceived susceptibility to, and severity of a disease, combined with lower levels of vaccine confidence, were associated with spending more time searching for information which could result in misinformation and adversely affect decision making (Clarke et al., 2019). Low levels of vaccine confidence combined with decreased concerns about vaccine preventable diseases are issues best addressed by a well-informed healthcare professional, however this cannot happen when therapeutic relationships are affected by poor education and communication.

In summary, this review has demonstrated the important role of healthcare professionals and their need to receive education on the beliefs, decision-making processes, and influences on vaccine hesitant parents. Evidence suggests that lack of knowledge and sensitivity of healthcare professionals has seriously, and in some cases irretrievably, affected the therapeutic relationship (Costa-Pinto et al., 2018; Saada et al., 2015). Once this relationship has broken down, parents sought information elsewhere. Meeting the needs of vaccine hesitant parents can be both confronting and challenging for healthcare professionals and with inadequate education, therefore, it is unsurprising that parents report unsatisfactory communication and therapeutic relationships. Immunisation education must become a core focus to fully inform healthcare professionals involved in immunisation (Attwell et al., 2018; Smith et al., 2020).

### 3.3 Alternative influences on vaccine decision making

This theme included several aspects which played a part in vaccine decision-making. These included friends and family, complimentary and allied medical practitioners, religion, social media and the internet, conspiracy theories and salutogenic parenting (Table 4). Conflicting information existed about the influence of friends and family members on vaccine choices (Costa-Pinto et al., 2018; Peretti-Watel et al., 2019; Rumetta et al., 2020). One UK study reported that 68% of parents reported their input did not influence their vaccine choices (Clarke et al., 2019). However, other studies reported the important influence of peers and significant others on vaccination attitudes and decision making (Syiroj et al., 2020). One Malaysian based study linked the lack of trust in healthcare professionals with increasing reliance on significant others to assist in their decision making (Peretti-Watel et al., 2019; Rumetta et al., 2020). Despite conflicting evidence about the influence of friends and family across the globe, it was apparent that when healthcare professionals were not considered a valued and trusted source of information, other sources took on a more significant and valued role. This role is dependant upon what is most accessible and valued in each country.

The use of complimentary and allied medical practitioners continues to be reported in recent studies, however, whilst complimentary and allied medical practitioners were associated with vaccine decision making, evidence suggests that it is not a direct cause of vaccine hesitancy, however, may coexist with vaccine rejection (Dube et al., 2016; Attwell et al., 2018; Costa-Pinto et al., 2018; Helps et al., 2019; Swaney and Burns, 2019; Syiroj et al., 2019). Parents included in the study by Attwell et al. (2018), reported using complimentary and allied medical practitioners as a method of supporting healthy children and were considered complimentary to parenting styles.

Social media has become a trusted source of information however, exposure has been associated with an increased risk of parents rejecting immunisation. Studies included in this review confirm that parents who elected to delay immunisation, often did so because of influences within their social media network (Dube et al., 2016; Costa-Pinto et al., 2018; Tustin et al., 2018; Duchsherer et al., 2020). An Italian study reported social media was a factor that influenced vaccine-related decisions (Diaz Crescitelli et al., 2020). Similarly, two Canadian studies reported higher odds of perceiving vaccines as unsafe, after searching social media sites (Tustin et al., 2018). In France, considerable mistrust of healthcare professionals and official vaccine information, has been associated with an increased reliance on unofficial internet sites, thereby increasing exposure to inaccurate information (Diaz Crescitelli et al., 2020). This was supported by a content analysis of social media groups conducted in the USA. Vaccine hesitant parents who posted on these pages, cited their main sources of information as social media, anti-vaccination documentaries and anti-vaccination websites (Bradshaw et al., 2020; Jenkins and Moreno, 2020).

In contrast, a study by (Giambi et al., 2018) reported that only 33% considered the internet to be reliable and, therefore, did not use it as a source of immunisation information. In the UK, it has been reported that information obtained by parents from the internet supported vaccination on most occasions (57%), suggesting that it is likely to have a positive effect on vaccine choices (Clarke et al., 2019). Overall, it is evident that social media is influential in parental vaccine decision-making, and in most cases, this influence heightens parental vaccine hesitancy. In a digital world, little can be done to discourage parents seeking information online, however, more should be done to ensure the accuracy of data in this space. This is of importance during the Covid-19 pandemic, when anxiety associated with vaccines appears to be increasing.

Studies included in this review have reported multiple belief systems and demonstrated distrust in both vaccine contents and pharmaceutical companies in general (Dube et al., 2016; Koski and Holst, 2017, 2018; Gidengil et al., 2019; Helps et al., 2019; Rossen et al., 2019; Swaney and Burns, 2019; Diaz Crescitelli et al., 2020; Rumetta et al., 2020). Beliefs in debunked studies also persisted throughout the literature as do inaccurate theories on vaccine contents (Helps et al., 2019). Several studies reported that parents want more information on vaccine contents and greater clarity on the rationale and timing of vaccine schedules (Rozbroj et al., 2020; Tomljenovic et al., 2020). The literature confirmed that conspiracy theories continue to influence vaccine choices, and vaccine hesitant parents are inundated with inaccurate information. Clear explanations by healthcare professionals on these issues may resolve this confusion and allow parents to make better choices.

Religious beliefs ceased to be a valid reason for vaccine exemption in Australia in 2015, however, continue to be a reason for vaccine refusal in several other countries including USA, Malaysia and Indonesia (Syiroj et al., 2019; Rumetta et al., 2020). Eighteen states in the USA still allow non-medical reasons, including religious and philosophical reasons for vaccine exemptions (Saada et al., 2015). Religion was also a cause of vaccine hesitancy in Malaysia and Indonesia; however, this was largely based on misinformation (Syiroj et al., 2019; Rumetta et al., 2020). Studies by Rumetta et al. (2020) and Syiroj et al. (2019) cited beliefs by the Islamic community that vaccines contained pork products which were Haram or forbidden. Whilst vaccines have been approved by Islamic Scholars and the WHO, and been given the certification of Halal status, mistrust persisted. This combined with a deep belief in natural immunity, concerns of safety and distrust in vaccines accounted for a large proportion of vaccine hesitancy in predominantly Islamic countries.

Salutogenic parenting is an approach to parenting which focusses on health and wellbeing rather than on factors which cause disease (pathogenesis) (Merriam-Webster.com). It is concerned with the relationship between health, stress, and coping and often includes long term breastfeeding, organic eating, avoiding toxins, reduced screen time, exercise, and fresh air. Many studies report parents using this form of parenting, were encouraged by a desire for natural living, healthy eating and reduced exposure to chemicals (Dube et al., 2016; Koski and Holst, 2017; Danchin et al., 2018; Helps et al., 2018; Gidengil et al., 2019; Helps et al., 2019; Peretti-Watel et al., 2019; Rumetta et al., 2020; Bradshaw et al., 2020; Diaz Crescitelli et al., 2020; Swaney and Burns, 2019). Several studies expressed the use of salutogenic parenting as complimentary to living, however, associated with this lifestyle choice was a significant fear of vaccine side effects and a disregard for the risks associated with vaccine preventable diseases (Swaney and Burns, 2019). Whilst there was evidence to suggest that salutogenic parenting co-existed with vaccine hesitancy, there was no indication in the literature, to suggest that it was a direct cause of it.

## 4 Discussion

The findings revealed that healthcare professionals have a critical role to play in information provision, education, and promotion of immunisation. Analysis of the literature suggests that the information seeking behaviour of pregnant women and parents is a significant factor in vaccine hesitancy. Whilst healthcare professionals are an important source of information, conversations with vaccine hesitant parents can be challenging and healthcare professionals report feeling inadequately prepared to promote and provide antenatal and childhood immunisation (Clarke et al., 2019; Facciola et al., 2019; Van Buynder, Van Buynder, Menton, Thompson, and Sun, 2019; Kennedy, 2020; Lama et al., 2020; Smith et al., 2020;). This review also reveals that education and support in vaccine decision making is best provided in pregnancy, and midwives are well placed to provide this (Danchin et al., 2018; Rosso et al., 2020). However, recent studies identify that midwives are both under-educated and under prepared for the role and there is a need for further education at an undergraduate level (Attwell et al., 2018; Smith et al., 2020).

Vaccine safety concerns have been shown to be a major influence on vaccine refusal (Danchin et al., 2018). Additionally, concerns about vaccine safety, result in considerable anxiety amongst parents with fear of reactions and long-term side effects. This is one area of concern which should be addressed as early as possible in pregnancy and parenting but before alternative sources of information are sought. Conspiracy theories are common on the internet and social media and vaccine safety is a prime focus of many antivaccination books and websites. With so much inaccurate information available to parents, it is the responsibility of healthcare professionals to address these concerns as early as possible in pregnancy to prevent the acquisition of misinformation (Ben Natan et al., 2017).

Addressing the perception of relative risk is difficult when therapeutic relationships with vaccine hesitant parents are strained (Omer et al., 2019). Additionally, it could be argued that few parents have firsthand experience of vaccine preventable diseases. Polio is unknown in most developed countries, as are diphtheria and tetanus (WHO, 2021). Measles has resurfaced recently in both Australia and Samoa largely due to pockets of low immunisation uptake. Despite the potential morbidity and mortality associated with Measles, it is often considered by parents to be a minor childhood illness (Swaney and Burns, 2019; Craig et al., 2020;). Multiple factors influence the decision to accept or reject vaccines based on perceived safety concerns including false reports of autism links in the case of measles. Despite significant evidence to debunk this concern, fears persist (Dawson and Apte, 2015).

Complementary and allied medical practitioners are a diverse group of practitioners which includes chiropractic, naturopathy and other modalities not usually offered by traditional medicine. This group of practitioners have previously been associated with having a negative impact on immunisation uptake (Wardle et al., 2016; Chow et al., 2017). Literature included in this review suggests that alternative influences such as complimentary and allied medicine and religion, have limited influence in an Australian setting (Attwell et al., 2018; Rumetta et al., 2020; Syiroj et al., 2019). However, there is conflicting evidence about the influence of friends and family internationally. Few studies exist within an Australian setting that have evaluated its impact on vaccine uptake. However, friends and family members remain a significant influence in countries such as Malaysia and Indonesia (Syiroj et al., 2019; Rumetta et al., 2020). Other factors which continue to influence vaccine decision making include social media which has become a trusted source of information for many parents (Clarke et al., 2019).  Exposure to this medium is associated with an increased risk of parents questioning the safety of immunisation (Atkinson et al., 2016; Vrdelja et al., 2018). Additionally, social media sites have been the subject of recent research and their role in supporting vaccine hesitancy is becoming evident (Bradshaw et al., 2020; Duchsherer et al., 2020; Jenkins and Moreno, 2020). However, the role of social media is not fully understood, and this is an area in need of further research.

Conspiracy theories have existed for almost as long as immunisation, and continue to exist in multiple forms (Kennedy, 2020; Kumar et al., 2016; Mendel-Van Alstyne et al., 2018). Parents who hold strong beliefs influenced by misinformation are some of the most vaccine hesitant. Whilst debunking conspiracy theories is complex, improved healthcare provider education and a consistent approach may assist in addressing this. Australia has adopted a consistent approach by refusing to accept non-medical exemptions to vaccination, however, this is not the case in other countries. Eighteen states in the USA still allow non-medical reasons for exemption, including religious and philosophical reasons for vaccine exemptions (Olive et al., 2018). This is not a consistent approach, nor does it support vaccine confidence.

The desire for a more natural lifestyle, often described as salutogenic parenting, has been seen to co-exist with vaccine hesitancy. Parents have reported using salutogenic parenting as a means of supporting the immunity of an unimmunised child (Schanfarber, 2015; (Ward et al., 2017)). This is an area where healthcare professionals are well placed to address concerns, correct misinformation and support decision making which assumes that living a healthy lifestyle offers protection against vaccine preventable diseases.

A primary limitation of this review is the nature of vaccine hesitancy itself. It has been described as a context specific phenomenon (SAGE working group, 2020). Each country included in this review has its own immunisation guidelines, policies, and legislation to promote vaccine compliance. This means that articles may not be directly comparable because of vastly differing local conditions present in each country. However, by incorporating articles from multiple countries, this review has the broadest possible focus on vaccine hesitancy thereby informing health practice globally.

## 5 Conclusion

The focus of this review is decision making in vaccine hesitant pregnant women and parents. Findings suggest that vaccine decision making is a complex process which for some, continues throughout pregnancy and childhood. Primarily, vaccine safety concerns induce high levels of anxiety with parents seeking information from multiple sources including healthcare professionals, the internet, friends and family and social media. Additionally, studies report a degree of dissatisfaction in the attitude and information provided by healthcare professionals in general and whilst the importance of healthcare professionals was recognised in some articles; this was not always reflected in commentary by parents. Recent studies have identified that midwives, whilst a trusted source of information are underprepared for their role. There is a need for further education at the undergraduate level to adequately prepare them for their important role (Attwell et al., 2019; Smith et al., 2020). Concerns also persist about the adverse effects of vaccines, the influences of complimentary and allied medical practitioners, religion and salutogenic parenting, which continue to be prevalent in the literature.

## Funding

No funding was received for this review.

## Declaration of Competing Interest

The authors state that they have no conflict of interests
